# Relative Deprivation, Poverty, and Subjective Health: JAGES Cross-Sectional Study

**DOI:** 10.1371/journal.pone.0111169

**Published:** 2014-10-28

**Authors:** Masashige Saito, Katsunori Kondo, Naoki Kondo, Aya Abe, Toshiyuki Ojima, Kayo Suzuki, the JAGES group

**Affiliations:** 1 Department of Social Welfare, Nihon Fukushi University, Aichi, Japan; 2 Center for Preventive Medical Science, Chiba University, Chiba, Japan; 3 Department of Health Economics and Epidemiology Research, The University of Tokyo, Tokyo, Japan; 4 Department of Empirical Social Security Research, National Institute of Population and Social Security Research, Tokyo, Japan; 5 Department of Community Health and Preventive Medicine, Hamamatsu University School of Medicine, Shizuoka, Japan; 6 Department of Social Studies, Aichi Gakuin University, Aichi, Japan; Waseda University, Japan

## Abstract

To evaluate the association between relative deprivation (lacking daily necessities) and subjective health in older Japanese adults, we performed a cross-sectional analysis using data from the Japan Gerontological Evaluation Study (JAGES). The data were obtained from functionally independent residents aged ≥65 years from 24 municipalities in Japan (n = 24,742). Thirteen items in three dimensions were used to evaluate relative deprivation of material conditions. Approximately 28% of older Japanese people indicated that they lacked some daily necessities (non-monetary poverty). A two-level Poisson regression analysis revealed that relative deprivation was associated with poor self-rated health (PR = 1.3–1.5) and depressive symptoms (PR = 1.5–1.8) in both men and women, and these relationships were stronger than those observed in people living in relative poverty (monetary poverty). The interaction effect between relative deprivation and relative poverty was not associated with poor health. As a dimension of the social determinants of health, poverty should be evaluated from a multidimensional approach, capturing not only monetary conditions but also material-based, non-monetary conditions.

## Introduction

The association between poverty and health has been established. A number of studies have revealed that relative income poverty is significantly related to poor health [1]–[3]. However, this approach has limitations when attempting to capture the diverse and complex aspects of poverty. In reality, older people tend to have comparatively high-quality living conditions due to savings and property ownership, even if their income is low [4]. To overcome this limitation, poverty research has proposed the concept of relative deprivation in material conditions to reflect the multidimensional non-monetary aspects of poverty [5]–[15]. Townsend [5] developed 60 relative deprivation indices within 12 dimensions composed of items such as “Household does not have a refrigerator” and “Has not had a week’s holiday away from home in the last 12 months,” and found that poverty in the United Kingdom was more extensive than generally believed or officially reported. The Europe 2020 strategy has adopted the concept of relative deprivation as a material dimension of social exclusion and has set the elimination of severe material deprivation as a goal for the next decade [16].

In investigating relationships between poverty and health, some studies have applied a social indicator approach, such as the Carstairs deprivation score or Townsend deprivation index, to include unemployment rate or proportion of non-car ownership. These previous studies found that relatively deprived areas were associated with standardized mortality rates [17]–[20], cancer mortality rates [21], suicide rates [22], coronary heart disease [23], dental caries [24], number of sound teeth remaining [25], and depression symptoms [26]. However, there have been few studies analyzing the relationship between an individual’s relative deprivation and his or her health. Some studies have shown an association between deprivation in living conditions and poor health [14], low levels of satisfaction with life [27], and poor social support [28]. Furthermore, no studies investigating the different associations between relative deprivation, relative poverty, and health have been conducted, although it has been suggested that people who live in relative deprivation have different characteristics than those living in relative poverty [9], [13], [15]. In order to clarify the relationships between poverty and health, it is important to identify the aspects of poverty that are the strongest predictors of health.

In addition, there have been few studies focusing on relative deprivation among older adults [8], [15]. Previous studies comparing younger and older people found that both the prevalence and depth of deprivation were more severe in younger people or single parents of working age [4], [13], [29]. However, from a life course perspective, the impact of relative deprivation on health should be evidenced in older people because the influence of poverty on health may accumulate over time. Finally, with the exception of a study that analyzed 131,335 people in 11 countries [10], most of the previous related research has been conducted on a relatively small scale. Analysis of relative deprivation should be performed with a large sample in order to derive robust findings, as few cases fall into each of the deprivation indices.

The present study posed three research questions: (1) is relative deprivation associated with poor health, even after monetary poverty is controlled for; (2) is the interaction effect between relative deprivation and relative poverty significantly associated with poor health, and (3) do older people with social support have good health, even if they are relatively deprived? In social psychology and sociology, the concept of relative deprivation has also been used to measure psychosomatic stress related to complaints or dissatisfaction based on comparisons with their reference groups [30]–[38]. However, our focus on relative deprivation is based on Townsend’s definition used in the poverty and social policy research described above.

## Methods

### Study samples

We used data from the Japan Gerontological Evaluation Study (JAGES), which was cross-sectional in design. JAGES was a postal survey of 112,123 people aged ≥65 who were randomly selected from the older residents of 31 municipalities in Japan. Data were collected from August 2010 to January 2012, with a response rate of 66.3%. For our study, we included 24,742 respondents from 24 municipalities who answered a relative deprivation questionnaire. The average age of the respondents was 74.6 years [standard deviation (SD) = 6.4] and 54.1% were women. Our study protocol and questionnaire procedures were approved by the Ethics Committee in Research of Human Subjects at Nihon Fukushi University. Written informed consent was assumed with voluntary return of the questionnaire.

### Dependent variables

We used self-rated health and depressive symptoms as indicators of subjective health. Self-rated health and depressive symptoms have been found to be highly valid predictors of mortality, regardless of other medical, behavioral, or psychosocial factors [39], [40]. Self-rated health was measured using the question “How do you feel about your current health status: excellent, good, fair, or poor?” Responses were recoded into dichotomous response variables (excellent/good or fair/poor). Depressive symptoms were assessed using the short version of the Geriatric Depression Scale (GDS-15), which was developed for self-administration in the community using a simple yes/no format [41], [42]. The validity and reliability of this scale have also been confirmed for Japanese older people, and it is often used in Japanese surveys [43], [44]. According to Sheikh et al. [42], scores of ≥5 on the GDS-15 indicated the presence of depressive symptoms of mild to severe depression. Our data showed approximately 30% of respondents had depressive symptoms. It is consistent with preceding Japanese studies [43], [44].

### Independent variables: relative deprivation and relative poverty

The indicators of relative deprivation used in preceding studies differ, because the standard living condition or decent life varies by culture and times. In reference to preceding research including Japanese studies [5]–[8], [10]–[12], [14], we evaluated thirteen indices that equated “lack of daily necessities,” “lack of living environment,” and “lack of social life due to economic reasons” with a low standard of living. Although lack of access to medical and health care services is another important element of standard of living, this was excluded from our deprivation indices because we assumed it to be directly reflected in poor health. On the other hand, we included experience of cutoff of essential services as a typical condition of lacking decent life, which is also used as a measure of social exclusion in a Japanese study [45].

Indicators of “lack of daily necessities” included having no television, refrigerator, air conditioner, microwave oven, or water heater. “Lack of living environment” indicators included having no private toilet, kitchen, or bathroom in the house and having a dining room that was not separate from the bedroom. “Lack of social life due to economic reasons” indicators included having no telephone or formal dress, being absent from family celebrations and events during the previous year due to economic reasons, and having essential services such as water, electricity or gas, cut off in the previous year (except in cases of forgetting to make a payment).

Relative poverty was defined as an income of less than half of the median annual equivalent income in the National Survey of Family Income and Expenditure in Japan [46]; the threshold was 1.49 million Japanese yen. This is the definition of relative poverty accepted by the Organisation for Economic Co-operation and Development and is conceptually based on the relative approach of the Luxembourg Income Study [47]. We used annual pre-tax household income. For each response, we calculated the equivalent household income by dividing income by the square root of the number of household members. Responses were categorized into three groups: poverty (28.2%), non-poverty (51.7%), and unknown (20.1%).

### Covariates

We used several control variables at the individual level: sex, age, educational attainment (to represent ascribed and achieved statuses), medical treatment (to represent recent physical condition), self-recognition of forgetfulness (to represent prodrome of dementia), and social support (to represent a buffer between poverty and health). Medical treatment was determined by asking “Are you currently receiving any medical treatment?” Self-recognition of forgetfulness was measured by asking “Do people around you notice your forgetfulness, for example, by telling you that you often ask the same thing?” Social support was measured using two questions representing emotional and institutional support: “Do you have someone who listens to your concerns and complaints?” and “Do you have someone who looks after you when you are sick and have to stay in bed for a few days?” Responses of “no” or “nobody” to both questions indicated an absence of social support.

In our data set, individuals were nested within each municipality. Previous studies reported significant associations between individual health and regional characteristics such as social capital and income inequality [48], [49]. We used the proportion of older people residing in the area (%), population density in inhabitable areas (1,000 person/km2), and the proportion of persons receiving public assistance (‰) for characteristics at the municipal level. These variables were based on 2010 census and government data for Japan. The distribution of these variables is shown is Table 1.

**Table 1:** Distribution of control variable.

Variable	Category	Total	Men	Women
**Individual** (n = 24,742)				
Sex	men	45.9	-	-
	women	54.1	-	-
Age	65–69	25.4	26.9	24.0
	70–74	29.1	29.5	28.7
	75–79	23.3	23.2	23.4
	80–84	14.1	13.5	14.7
	85 -	8.1	6.8	9.2
Education	>9	47.6	51.8	44.1
	= <9	49.9	46.5	52.9
	unknown	2.4	1.7	3.0
Marital status	married	69.0	84.0	56.0
	divorced	22.6	8.6	34.5
	separated	3.2	2.6	3.8
	never married	2.1	1.9	2.3
	unknown	3.1	2.9	3.4
Disease and/or impairment	no	22.4	24.1	20.8
	yes	68.5	67.6	69.3
	unknown	9.1	8.3	9.9
Self recognition of forgetfulness	no	79.3	79.5	79.1
	yes	16.8	16.5	17.1
	unknown	3.9	4.1	3.8
Social supports	present	85.6	84.4	86.7
	absent	8.1	9.9	6.5
	unknown	6.3	5.7	6.8
**Municipality** (n = 24)				
Proportion of older people	mean (SD)	24.4 (5.2)	-	-
Population density[1000 p/km^2^]	mean (SD)	1.70 (1.36)	-	-
Proportion of person receivingpublic assistance[‰]	mean (SD)	8.5 (10.7)	-	-

### Statistical analysis

First, we confirmed the distribution of the relative deprivation index and calculated crude odds ratios for subjective health. Second, we applied two-level Poisson regression analysis of random-intercept and fixed-slope models to assess the relationship between relative deprivation and self-rated health and depressive symptoms, adjusting for individual factors and municipal-level covariates (level 1: 24,742 individuals, level 2: 24 municipalities). We adopted multilevel modeling in order to control for intraclass (municipal level) cluster correlation. We also examined the interaction effect of relative deprivation and relative poverty. Individual and municipality fixed parameters were converted to prevalence ratios (PR) with a 95% confidence interval (95%CI). Finally, we calculated the proportion of poor health among deprived people with social support. We used the computer software, STATA 12.1 for all analyses.

## Results

Table 2 showed that 1.6% to 8.4% of respondents lived in deprived conditions, as defined by the study parameters. A higher percentage of respondents did not have a dining room separate from the bedroom (14.9%). Univariate analysis showed that ORs for respondents living in deprived conditions, according to each relative deprivation index, were approximately 1.3–2.5 times higher for fair/poor self-rated health and 1.7–4.1 times higher for depressive symptoms than respondents who did not live in relative deprivation. In particular, the crude ORs for having essential services cut off and absence from family celebrations and events were relatively high. Relative deprivation scores also showed that respondents who were deprived of one item (14.3%) and two or more items (13.6%) were more likely to report fair/poor self-rated health [OR = 1.61 (95%CI: 1.48–1.75) and OR = 1.86 (95%CI: 1.70–2.02), respectively] and depressive symptoms [OR = 1.93 (95%CI: 1.77–2.10) and OR = 2.57 (95%CI: 2.35–2.81), respectively].

**Table 2:** Distribution of relative deprivation index.

					Crude odds ratio (95% CI)	
Dimension	Item	Category	n	%	self-rated health(1 = fair/poor)	depressive symptom(1 = present)
Lack of daily necessities dueto economic reasons	no television	no	23,594	97.6	1.00	1.00
		yes (+)	592	2.4	1.47 (1.22–1.77)	2.25 (1.87–2.72)
	no refrigerator	no	23,781	98.3	1.00	1.00
		yes (+)	405	1.7	1.32 (1.05–1.66)	1.67 (1.32–2.11)
	no air conditioner	no	22,823	94.4	1.00	1.00
		yes (+)	1,363	5.6	1.62 (1.44–1.83)	2.23 (1.97–2.52)
	no microwave oven	no	23,315	96.4	1.00	1.00
		yes (+)	871	3.6	1.62 (1.40–1.88)	1.88 (1.61–2.19)
	no water heater	no	23,213	96.0	1.00	1.00
		yes (+)	973	4.0	1.63 (1.41–1.87)	2.14 (1.85–2.48)
Lack in living environment	private WC	yes	22,606	93.5	1.00	1.00
		no (+)	1,580	6.5	1.40 (1.24–1.57)	1.78 (1.58–2.01)
	private kitchen	yes	22,260	92.0	1.00	1.00
		no (+)	1,926	8.0	1.43 (1.29–1.59)	1.79 (1.60–2.00)
	private bathroom	yes	22,153	91.6	1.00	1.00
		no (+)	2,033	8.4	1.40 (1.27–1.56)	1.83 (1.64–2.04)
	dining room separated from bedroom	yes	20,585	85.1	1.00	1.00
		no (+)	3,601	14.9	1.48 (1.37–1.61)	1.81 (1.67–1.97)
Lack of social life dueto economic reasons	no telephone	no	23,229	96.0	1.00	1.00
		yes (+)	957	4.0	1.52 (1.32–1.76)	2.25 (1.94–2.60)
	no ceremonial dress	no	23,644	97.8	1.00	1.00
		yes (+)	542	2.2	1.53 (1.27–1.85)	1.92 (1.58–2.34)
	absence from relative's ceremonial occasions	no	21,952	93.4	1.00	1.00
		yes (+)	1,549	6.6	2.47 (2.22–2.76)	3.27 (2.91–3.67)
	cut-off of essential services in the past year	no	23,509	98.4	1.00	1.00
		yes (+)	388	1.6	2.09 (1.70–2.59)	4.10 (3.27–5.14)
Number of relative deprivation index		none	16,812	72.0	1.00	1.00
		1	3349	14.3	1.61 (1.48–1.75)	1.93 (1.77–2.10)
		2	916	3.9	2.01 (1.74–2.32)	2.68 (2.31–3.12)
		3	480	2.1	2.04 (1.67–2.48)	2.89 (2.36–3.52)
		4	1109	4.8	1.61 (1.40–1.85)	2.04 (1.77–2.36)
		5	271	1.2	2.30 (1.77–2.98)	3.49 (2.66–4.58)
		> = 6	401	1.7	1.75 (1.40–2.19)	2.92 (2.33–3.67)
		> = 2	3177	13.6	1.86 (1.70–2.02)	2.57 (2.35–2.81)

(+) is related to relative deprivation.

Table 3 shows the associations between subjective health and a combination of relative deprivation and relative poverty. The proportion of respondents with poor health was high with respect to “poverty and deprivation,” “deprivation only,” “poverty only,” and “no deprivation or poverty.” Odds ratios for respondents living in poverty and deprivation (two or more deprivation items) were 2.51 (95%CI: 2.21–2.86) and 3.53 (95%CI: 3.10–4.02) times higher for fair/poor self-rated health and depressive symptoms, respectively, than ORs for respondents with no deprivation or poverty.

**Table 3:** Combination of relative deprivation and poverty.

		Self-rated health		Depressive symptom	
	n (%)	fair/poor %	Crude OR(95%CI)	present %	Crude OR(95%CI)
No deprivation or poverty	10,241 (53.7)	16.9	1.00	21.0	1.00
Poverty only	3,987 (20.9)	23.3	1.50(1.37–1.64)	29.0	1.54(1.41–1.68)
Deprivation only ( = 1)	1,334 (7.0)	23.1	1.48(1.29–1.70)	32.1	1.78(1.56–2.03)
Deprivation only (> = 2)	893 (4.7)	25.4	1.68(1.43–1.97)	36.4	2.16(1.85–2.52)
Poverty and deprivation ( = 1)	1,305 (6.8)	31.4	2.26(1.98–2.57)	43.2	2.86(2.51–3.26)
Poverty and deprivation (> = 2)	1,300 (6.8)	33.8	2.51(2.21–2.86)	48.4	3.53(3.10–4.02)

Table 4 shows the results of a two-level Poisson regression analysis. Random effects showed that municipal-level variance in each model was smaller than that in the null model. This means that part of the municipal-level variance was explained by the individual- and municipal-level variables in the model. Fixed effects showed similar associations for both genders. Respondents with low educational attainment, no social support, under medical treatment, and prodrome of dementia tended to have poor subjective health, although relationships between age and municipal level variables were not consistent.

**Table 4:** Association of subjective health and relative deprivation by two-level Poisson regression analysisa).

	Self-rated health (fair/poor)				Depressive symptom (present)			
	Men		Women		Men		Women	
	*PR*	(95%CI)	*PR*	(95%CI)	*PR*	(95%CI)	*PR*	(95%CI)
*FIXED EFFECTS*								
***Individual level***								
Age (ref.: 65–69)								
70–74	1.06	(0.94–1.19)	1.09	(0.97–1.23)	0.92	(0.83–1.02)	1.00	(0.90–1.11)
75–79	1.24***	(1.11–1.40)	1.29***	(1.15–1.46)	1.03	(0.93–1.15)	0.98	(0.88–1.10)
80–84	1.31***	(1.15–1.49)	1.49***	(1.31–1.70)	0.95	(0.84–1.08)	1.02	(0.90–1.15)
85 -	1.23*	(1.05–1.45)	1.54***	(1.33–1.79)	0.95	(0.81–1.13)	1.15*	(1.00–1.33)
Education (ref.: >9 years)								
= <9 years	1.07	(0.98–1.16)	1.19***	(1.09–1.29)	1.16***	(1.07–1.25)	1.09*	(1.01–1.18)
Marital status (ref.: married)								
divorced	0.99	(0.86–1.13)	0.83***	(0.76–0.90)	1.23**	(1.09–1.39)	1.03	(0.95–1.12)
separated	1.22	(0.99–1.51)	0.91	(0.75–1.10)	1.37**	(1.14–1.65)	1.10	(0.92–1.31)
never married	1.17	(0.90–1.53)	0.92	(0.72–1.19)	1.29*	(1.03–1.62)	0.98	(0.77–1.26)
Disease and/or impairment(ref.: no)								
yes	4.76***	(4.05–5.60)	3.90***	(3.34–4.56)	1.33***	(1.21–1.46)	1.28***	(1.16–1.41)
Self recognition of forgetfulness(ref.: no)								
yes	1.47***	(1.34–1.61)	1.65***	(1.52–1.79)	1.71***	(1.57–1.86)	1.83***	(1.69–1.98)
Social supports(ref.: present)								
absent	1.37***	(1.21–1.54)	1.58***	(1.39–1.79)	1.57***	(1.41–1.75)	1.84***	(1.64–2.07)
Relative poverty (ref.: non-poverty)								
poverty (< ¥ 1.49 million)	1.25***	(1.11–1.40)	1.13*	(1.01–1.26)	1.34***	(1.20–1.50)	1.24***	(1.11–1.37)
Relative deprivation score (ref.: none)								
1	1.19*	(1.03–1.37)	1.31***	(1.15–1.50)	1.45***	(1.27–1.65)	1.36***	(1.19–1.55)
> = 2	1.34***	(1.16–1.54)	1.27**	(1.10–1.46)	1.62***	(1.42–1.85)	1.43***	(1.25–1.64)
Interaction Effect								
poverty×deprivation (1)	1.08	(0.87–1.34)	0.95	(0.78–1.17)	1.03	(0.84–1.26)	1.00	(0.82–1.22)
poverty×deprivation (> = 2)	1.01	(0.82–1.25)	1.06	(0.86–1.31)	0.94	(0.77–1.14)	1.01	(0.82–1.23)
***Municipal level***								
Proportion of older people [1%]	1.00	(0.99–1.01)	0.99	(0.98–1.00)	1.01*	(1.00–1.02)	1.02**	(1.01–1.03)
Population density [1000 p/km^2^]	0.96**	(0.94–0.99)	0.94***	(0.92–0.97)	1.03*	(1.00–1.06)	1.01	(0.99–1.04)
Prop. of receiving public assistance [5‰]	1.03*	(1.00–1.06)	1.04**	(1.01–1.07)	0.96**	(0.93–0.99)	0.95**	(0.93–0.98)
*RANDOM EFFECTS* b)								
Municipality level variance(standard error)	.039 (.050)		.046 (.042)		.045 (.031)		.035 (.031)	

***p<.001 **p<.01 *p<.05 PR: Prevalence ratio.

a) Each estimated coefficient of “unknown” category was omitted in above table.

b) Random effect in null model:

SRH(men) = .147(SE = .034), SRH(women) = .197(SE = .037), GDS(men) = .093(SE = .027), GDS(women) = .101(SE = .026).

Relative deprivation was significantly associated with poor health, regardless of the status of relative poverty and other individual- and municipal-level characteristics. In male respondents with two or more deprivation items, rates of fair/poor self-rated health were 1.34 times (95%CI: 1.16–1.54) higher and rates of depressive symptoms were 1.62 times (95%CI: 1.42–1.85) higher than those observed in non-deprived individuals. Similarly, relative deprivation was associated with fair/poor self-rated health [PR = 1.27 (95%CI: 1.10–1.46)] and depressive symptoms [PR = 1.43 (95%CI: 1.25–1.64)] in women. Relative poverty was also related to self-rated health [men: PR = 1.25 (95%CI: 1.11–1.40); women: PR = 1.13 (95%CI: 1.01–1.26)] and depressive symptoms [men: PR = 1.34 (95%CI: 1.20–1.50); women: PR = 1.24 (95%CI: 1.11–1.37)]. The PRs for relative deprivation were comparatively higher than those for relative poverty. The interaction effect between relative poverty and relative deprivation for subjective health was not statistically significant.

**Figure 1:**
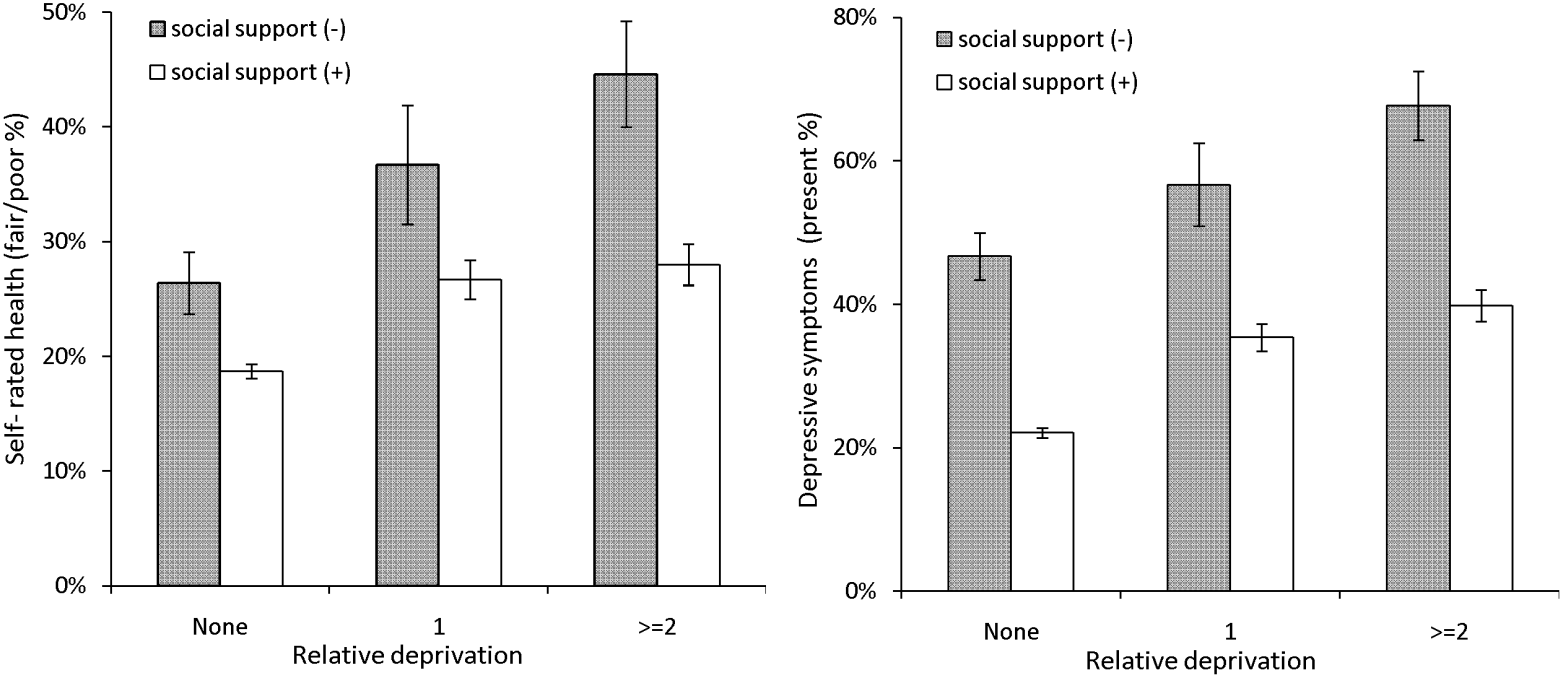
Proportion (95% Confidence Interval) of poor health in relation to relative deprivation and social support. Both figures show a low proportion of poor health in the presence of social support. Meanwhile, the proportion of those with poor health increases as the relative deprivation index score increases even with social support, indicating that social support does not fully cancel out the negative impacts of relative deprivation on health.

Figure 1 shows different associations between subjective health and relative deprivation according to social support. The proportion of respondents with poor health was remarkably lower in those with social support relative to those without. However, relative to non-deprived respondents with any level of social support, the proportions of respondents with poor/fair subjective health were remarkably higher in the relative deprivation groups (1 and ≤2, respectively).

## Discussion

It was previously thought that all Japanese people were middle class. However, recent surveys have shown that intergenerational inequality exists in Japan [50], and the number of older people on public assistance is increasing [51]. The World Health Organization stated that poverty and relative deprivation have a major impact on health and premature death [52]. On the other hand, most poverty research has been based solely on the concept of relative poverty (monetary poverty) since data on material and environmental poverty was severely limited in Japan.

The present study addressed the concepts of both relative poverty and relative deprivation through a large survey of older Japanese and analyzed the association between health and relative poverty and deprivation. Our results showed that relative deprivation and relative poverty were related to poor health, even after other variables were controlled for. Our results were consistent with preceding findings; Abe [14] found that relative deprivation is closely associated with poor self-rated health and the presence of depressive symptoms using national Japanese representative cross-sectional data of participants aged ≥20 years. In particular, our results suggest that the concept of relative deprivation could address a different aspect of poverty that is related to health but is not addressed by the concept of relative poverty. People who have overlapping multidimensional disadvantages are more likely to be socially excluded [9] and to experience premature death [53]. Our results also showed that the negative effects of relative poverty and deprivation on health are additive; people with both relative deprivation and poverty were more disadvantaged with respect to health, but the relationship was not multiplicative.

Our study adds new evidence regarding which elements of poverty have strong impacts on the health of older adults. An important finding is that relative deprivation has a stronger association with subjective health than relative poverty for both sexes. Some studies have revealed that people living in relatively deprived conditions experienced long-term, severe poverty throughout their life course [10], [11]. For example, Whelan et al. [10] reported that approximately 40% of persistent income poverty overlapped with lifestyle deprivation in a broadly uniform manner. Consequently, the concept of relative deprivation could capture severe and absolute poverty better than relative poverty, which is based on the distribution of income in society. This could mean that relatively deprived older people might tend to be more disadvantaged, even in good health.

Finally, similar to a preceding study using cross-sectional data from 5,624 women aged 2059 [28], our data confirmed that relative deprivation was associated with the absence of social support [15]. Moreover, our results showed that having social support of any form could mitigate some of the negative impacts of relative deprivation on health. However, it is important to note that even with social support, relatively deprived people have more disadvantages with respect to health than non-deprived people. Therefore, the effects of material and environmental deprivation on poor health cannot be explained only by the absence of social support. As shown in preceding study [30]–[38], relative deprivation might increase social stresses and anxieties while lowering self-efficacy by depriving a living standard most people in the society enjoy. Furthermore, unlike monetary poverty, poor standard of living such as relative deprivation might closely be related to unhealthy lifestyles including poor eating habit and nutrition and lack of access to healthcare and welfare services.

Compared to relative poverty, which is based on a simple indicator and is often used in international comparative studies, relative deprivation is composed of complex indicators and has limitations for use in comparative studies. In fact, most preceding studies have applied a consensual approach based on public opinion in creating and selecting daily necessities and basic needs indices [4], [6], [7], [10], [12]–[14]. As a result, each relative deprivation indicator was different among preceding studies, although they often reflected the characteristics of that nation and culture. However, relative deprivation could more accurately represent the phenomenon of poverty due to multidimensional living conditions than it does relative poverty. Although measurements of relative deprivation have been made in order to establish the poverty line, our results suggest that relative deprivation is also important for public health policy as it represents a dimension of the social determinants of health.

### Study limitations

The present study has some limitations. First, our relative deprivation indicators did not cover the full range of daily resources among older people in Japan. Although we included indicators used in preceding studies, the indicators should be more sophisticated. Second, while the overall response rate for our data was relatively high, the response rates among the lower income categories were comparatively lower [54]. Therefore, our findings may be underestimated because people living in serious poverty and deprivation may have been less likely to participate in our survey. Third, there is a possibility of selection bias at the municipal level since our data are not representative of the whole country. On the other hand, our subjects were randomly selected in each municipality, and it is important to note that we did perform a large-scale survey concerning non-monetary poverty among older people in more than one municipality. Further research should include longitudinal surveys to reveal whether a causal relationship between relative deprivation and health exits.

## Conclusion

Relative deprivation (non-monetary poverty) is an important element in poverty. To the best of our knowledge, this is the first study to investigate the association between health conditions and relative deprivation and poverty among older Asian adults. The results showed that relative deprivation has stronger associations with self-rated health and depressive symptoms than with relative poverty. There was an independent and additive association between relative deprivation and poverty with respect to subjective health, and the presence of social support may not fully mitigate the negative association between relative deprivation and health. Our results suggest that relative deprivation is one social determinant of health that the concept of relative poverty cannot address.
